# New *para*-hydrogen cold neutron source at the Budapest Research Reactor: Monte Carlo simulations

**DOI:** 10.1107/S1600576726001263

**Published:** 2026-03-26

**Authors:** Dmitrii Shapiro, Petr Konik, Mina Akhyani, Alexander Ioffe

**Affiliations:** a Jülich Centre for Neutron Science at Heinz Maier-Leibnitz Zentrum (MLZ) Lichtenbergstraße 1 85748Garching Germany; b Budapest Neutron Centre HUN-REN Centre for Energy Research, Konkoly Thege Miklós út 29-33 1121Budapest Hungary; c Department of Physics and Technology, Al-Farabi Kazakh National University, Al-Farabi Ave. 71, Almaty 050040, Kazakhstan; Technical University of Denmark, Denmark

**Keywords:** cold neutron moderators, neutronics, Budapest Research Reactor, *para*-hydrogen

## Abstract

The planned upgrade of the neutron guide system at the Budapest Research Reactor (BRR) provides an opportunity to enhance the performance of its cold neutron source. In this work, two replacement concepts for the existing liquid hydrogen disk moderator are investigated: (i) a box-shaped *para*-hydrogen (pH_2_) moderator and (ii) assemblies of low-dimensional (flat) pH_2_ moderators. Both designs are optimized using the curve of full and optimal sample illumination method and through Monte Carlo simulations tailored to the BRR geometry and the thermal neutron distribution. The box pH_2_ moderator is found to increase the cold neutron brightness by up to 2.2 times in the 3–7 Å wavelength range. Flat moderator assemblies, as either staircases or chessboards, provide an additional gain of about 20% at 2 Å, although their advantage diminishes at longer wavelengths due to the non-uniform thermal illumination of the reactor channel and the wavelength dependence of the optimal moderator length. These results demonstrate that pH_2_ moderators, particularly in low-dimensional geometries, can substantially enhance the performance of the upgraded BRR cold neutron source, with the achievable gains largely limited by the thermal neutron distribution within the existing moderator channel.

## Abstract 2

This paper explores the options for upgrading the cold neutron source at the Budapest Research Reactor.

## 1. Introduction

The Budapest Research Reactor (BRR) is a 10 MW tank-type research reactor with a beryllium reflector, moderated and cooled by light water, which was equipped with a cold neutron source in 2001 (Rosta *et al.*, 2002 ▸).

An upcoming upgrade of the BRR neutron guide system offers an opportunity to replace the existing cold neutron source with a more efficient one. While the current source uses a liquid hydrogen moderator, several alternatives have been evaluated within the framework of the BrightnESS project (Rosta *et al.*, 2018 ▸). These studies have demonstrated that the most promising option is the implementation of a *para*-hydrogen (pH_2_) low-dimensional moderator.

**Figure 1:**
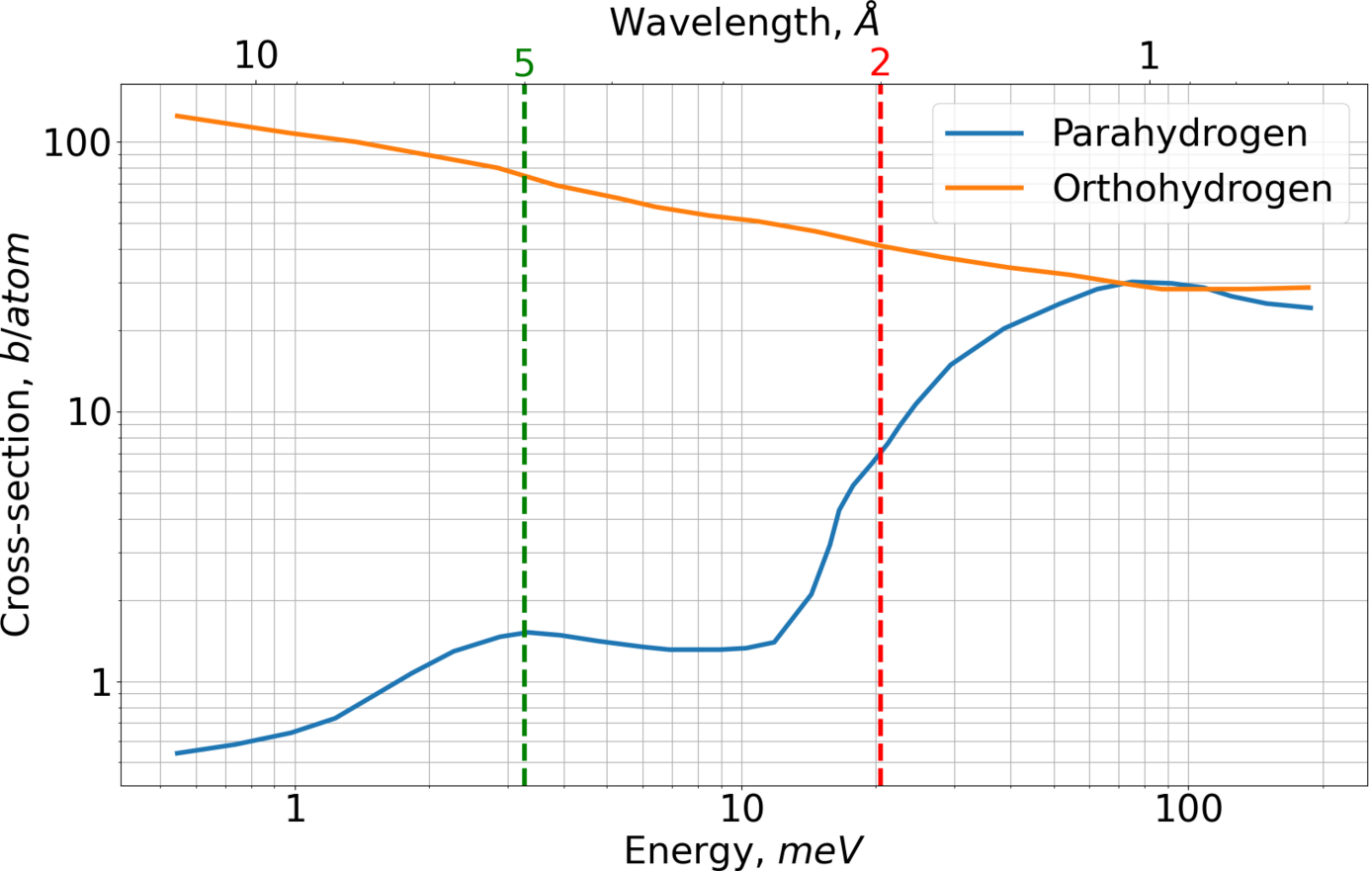
Scattering cross sections of *para*- and *ortho*-hydrogen. Vertical dashed lines correspond to the two wavelengths of 2 and 5 Å investigated in this paper. Data are taken from Watanabe (2003 ▸).

*Para*-hydrogen has remarkable and, in fact, unique neutron scattering properties. The first is the *para*–*ortho* conversion, an inelastic process in which a neutron is slowed down by 14.7 meV in a single collision. This means that thermal neutrons with energies of 20–35 meV can be converted to cold neutrons without requiring multiple scattering, and thus the large moderator volume normally needed for thermalization is unnecessary. The second notable property of *para*-hydrogen is its neutron scattering cross section, which is one to two orders of magnitude higher for thermal neutrons than the typical values of a few barns for cold neutrons, as can be seen in Fig. 1 ▸.

Taken together, these two properties enable the concept of a low-dimensional, or flat, moderator (Batkov *et al.*, 2013 ▸; Mezei *et al.*, 2014 ▸), which significantly increases the brightness of the emitted cold neutron beam. Thermal neutrons, with a mean free path of about 1 cm, are effectively converted into cold neutrons within 2–3 cm in a single collision. Cold neutrons, whose mean free path is 10–20 cm, then propagate along the moderator surface with relatively small losses. Such flat moderators have been developed at the European Spallation Source (ESS, Lund, Sweden), where they are expected to provide 2.5–3 times higher brightness compared with conventional voluminous hydrogen moderators (Zanini *et al.*, 2019 ▸). Other facilities, such as the Spallation Neutron Source (SNS, Oak Ridge, Tennessee, USA), have followed the same path (Gallmeier & Remec, 2022 ▸).

**Figure 2:**
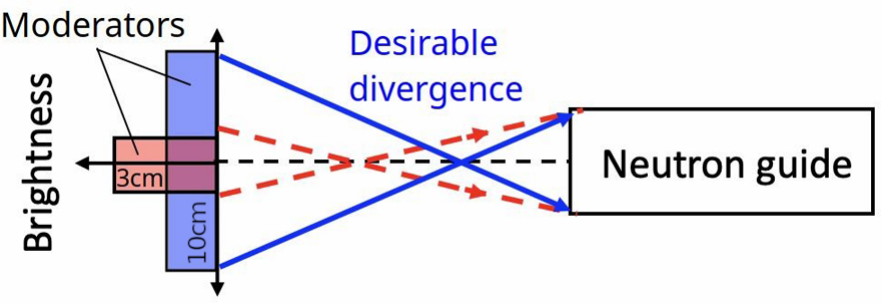
Reducing the width of the moderator increases brightness, but excessively narrow moderators under-illuminate the neutron guide, reducing instrument performance.

However, this higher brightness comes at the cost of reduced beam intensity due to the smaller moderator size – about a 20% reduction when coming from a 10 cm height to 3 cm (Zanini *et al.*, 2019 ▸). Moreover, the small moderator aperture leads to under-illumination of the neutron guide entrance and, consequently, to insufficient fulfillment of sample requirements for neutron phase space (Fig. 2 ▸). For bent neutron guides it also results in strong irregularities in the divergence profile of the outgoing beam. As a solution to these problems the concept of flat moderator assemblies was proposed (Ioffe *et al.*, 2025 ▸), which allows one to obtain both high brightness and high intensity, along with a large enough moderator aperture.

The present study deals with the investigation of the replacement of the current disk hydrogen moderator of the BRR with either a box *para*-hydrogen moderator or an assembly of flat *para*-hydrogen moderators. The structure of the paper is as follows. Section 2 details the BRR model and describes how Monte Carlo simulations have been carried out to evaluate the performance of various cold moderator configurations. Section 3 first investigates the properties of the box *para*-hydrogen moderator. Special attention is given to analyzing the phase space requirements of the BRR instrumentation suite [in particular the time-of-flight (TOF) spectrometer NEAT]. Using the curve of full and optimal sample illumination (COFSI) technique those requirements are re-formulated as constraints for the moderator optimization. Finally, two box moderator configurations, optimal for 2 and 5 Å neutron beams, are compared against the current moderator. The remainder of the section deals with the concept of flat pH_2_ moderator assemblies applied to the BRR. The limiting factors for their performance are identified and optimal solutions are compared with the current moderator as well as the optimal box moderators. The concluding Section 4 reiterates the results and outlines options proposed for the future BRR cold source upgrade.

## 2. Methodology

To simulate the performance of the moderators we have developed the BRR model using the Monte Carlo code *PHITS* (Niita *et al.*, 2006 ▸).

**Figure 3:**
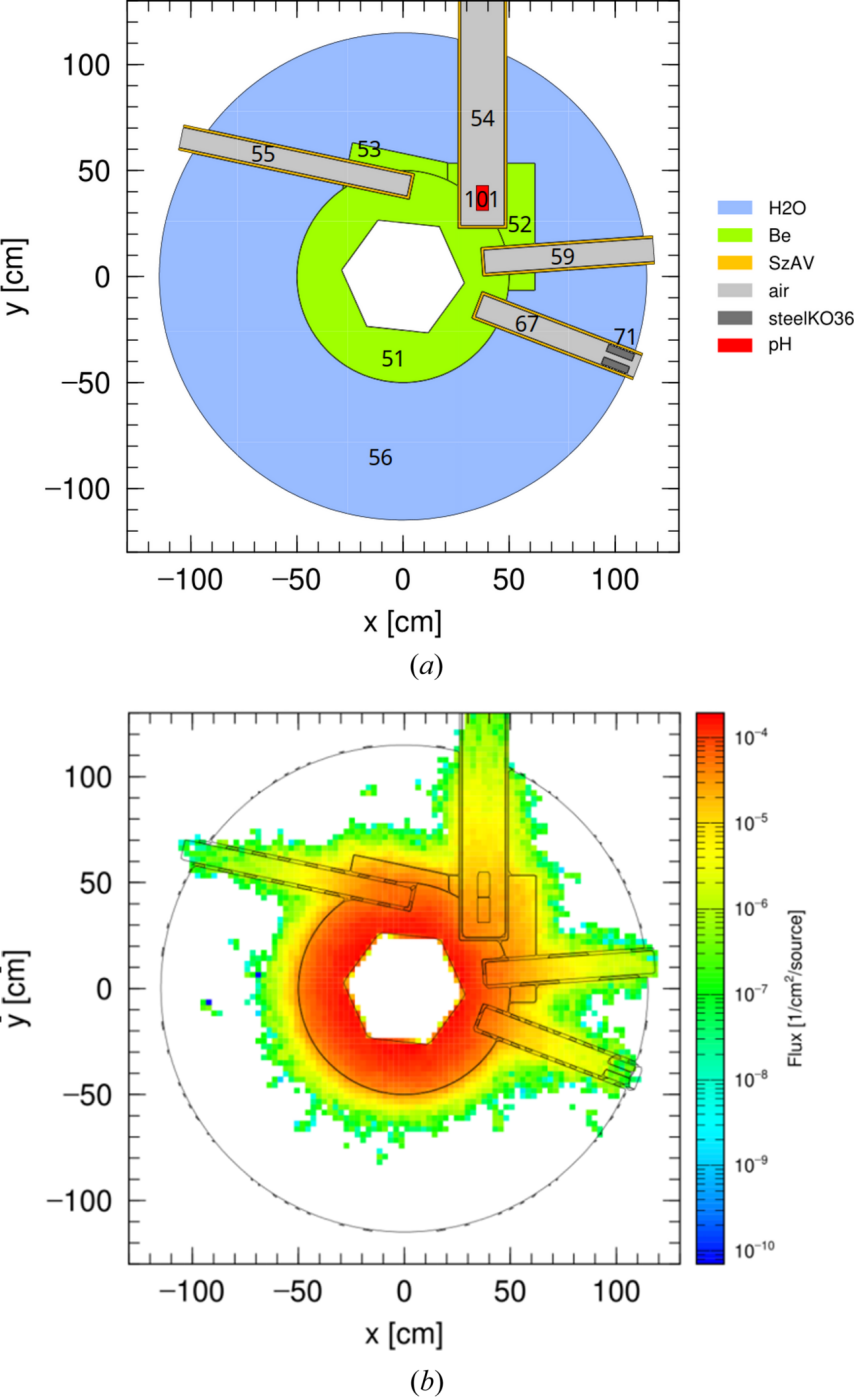
(*a*) Top view showing the geometry of the BRR model: horizontal cut in the plane of the channels. (*b*) The corresponding neutron flux map. Further details are provided in the text.

A top view of this model is shown in Fig. 3 ▸(*a*) and the distribution of the neutron flux is presented in Fig. 3 ▸(*b*). The hexagonal reactor core is surrounded by a beryllium tank (cell 51) with two additional beryllium boxes (cells 52 and 53), which in turn are enclosed by a light-water tank (cell 56). The cold moderator is shown as cell 101 and is located in the moderator channel labeled cell 54. Two additional neighboring channels, cell 55 and cell 59, are included because they perturb the illumination from the active zone towards the moderator channel. The channel denoted cell 67 contains a steel collimator (cell 71) and is used to reproduce the reactor core spectrum, as discussed below. All the channels are filled with air and their walls are made of the aluminium alloy SzAV-1 with a thickness of 1.3 cm. The thermal scattering library used in all simulations is JENDL-5 (Iwamoto *et al.*, 2023 ▸).

Since no reliable preliminary simulation data were available for the thermal neutron distribution in the moderator channel, an indirect modeling strategy was required. As the *PHITS* simulation package does not support direct treatment of uranium fission processes in reactor fuel, we approximate the neutron distribution by introducing a source term at the reactor core boundary, implemented as six uniform isotropic neutron sources placed on the side surfaces of the hexagonal prism [Fig. 3 ▸(*b*)].

**Figure 4:**
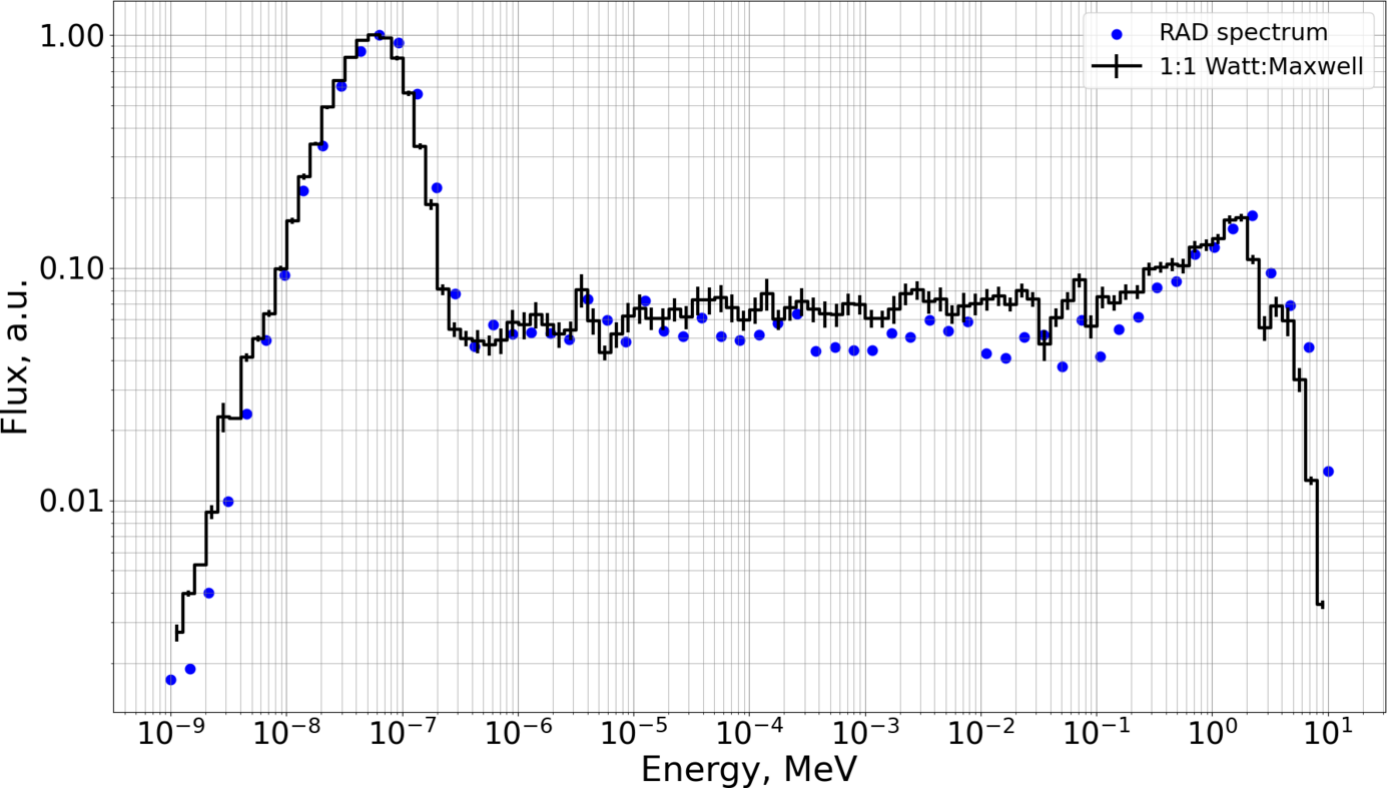
RAD spectrum obtained from *MCNP* model (blue dots) and from the BRR model with the source spectrum described by equation (3) with *x* = 0.5 (black line). Units are chosen in such a way that the maximum flux is unity.

This source term includes the fast neutrons originating from uranium fission, with an energy spectrum described by the Watt distribution *f*_W_(*E*):
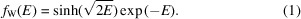
The source term also accounts for thermal neutrons produced from a fraction of fast neutrons moderated by light water within the reactor core. Their energy spectrum is described by the Maxwellian distribution *f*_M_(*E*, *T*):
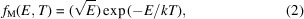
where *T* ≃ 320 K is the temperature of water in the primary cooling loop and *k* is the Boltzmann constant. A linear combination of equations (1) and (2) defines the energy distribution *f*(*E*, *T*) of the whole source,
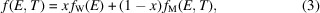
where *x* denotes the fraction of the Watt component. To determine *x* we used the spectrum of the white-beam neutron imaging station RAD, calculated with an *MCNP* (Forster & Godfrey, 1985 ▸) model of BRR, in which the neutrons were produced from uranium fission in the core (Kis *et al.*, 2015*b* ▸). RAD is located at the channel denoted cell 67, and the *MCNP* spectrum was calculated in the area inside the steel collimator (cell 71). We vary *x* in order to reproduce the same spectrum at the same place in the *PHITS* model. The best agreement is obtained for *x* = 0.5, corresponding to a 1:1 Watt–Maxwell ratio (Fig. 4 ▸).

To calculate the flux from the moderator we use a point tally T-Point positioned on the axis of the moderator channel 10 m downstream from the moderator exit surface. A detailed discussion of how the calculations are performed can be found in Appendix *A*[App appa].

To evaluate the performance of a moderator we select only ‘desired’ neutrons that scatter exclusively inside the moderator and we filter out other ‘undesired’ neutrons – direct neutrons from the reactor core and the neutrons scattered and re-scattered outside the moderator. This division was carried out using the particle event logger Counter. It can be thought of as a particle’s ‘quantum number’, which can change its value during different events – entering or exiting a cell or scattering inside a cell. A tally can be configured to register particles with a Counter value within a specific range or equal to a specific number.

For the simulations presented in this study, the Counter is configured so that each neutron scattering event occurring inside the moderator cell increments the Counter value by 1, while scattering inside any other cell leaves it unchanged. The T-Point tally is set to record only neutrons with a Counter value of not less than 1. Since T-Point is a next-event estimator, a neutron does not have to leave the moderator to be detected, so after scattering inside the moderator it is immediately recorded, whereas direct neutrons from the source and any prior collisions outside the moderator are excluded.

However, a particle keeps its Counter value after a collision, and subsequent scattering in non-moderator cells may be detected by T-Point erroneously. To prevent this, the Counter value is forcibly reset to 0 whenever a neutron exits the moderator cell. Thus, all collisions in non-moderator cells occur with a Counter value of 0 and are not recorded by T-Point. Note that multiple collisions inside the moderator are still tracked and counted properly.

The approach used here differs from the conventional method (Zanini *et al.*, 2019 ▸; Gallmeier *et al.*, 2010 ▸), which employs artificial collimators. Their use would necessitate thorough evaluation, since they may fail to eliminate ‘undesired’ neutrons completely or, conversely, they may remove the ‘desired’ ones. In contrast, our approach is non-invasive and straightforward to set up.

## 3. Optimization of pH_2_ moderator

### 3.1. Current cold neutron moderator at the BRR

**Figure 5:**
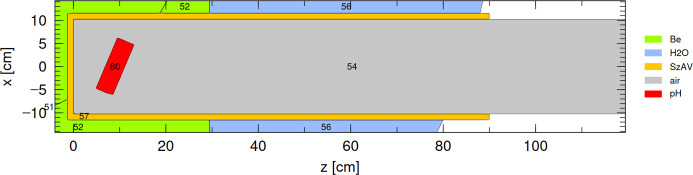
Top view of the moderator channel (cell 54) with a disk pH_2_ moderator (cell 80).

The current cold neutron moderator at the BRR is a disk with a radius of 6 cm and a thickness of 4 cm, rotated by 23°, which is shown as cell 80 in Fig. 5 ▸. Its 2.5 mm walls are made of the aluminium alloy SzAV-1. The disk is placed approximately 7 cm from the channel bottom and is filled with hydrogen at about 20 K. Since the *ortho*–*para* ratio has little effect on the brightness of this particular moderator (Rosta *et al.*, 2018 ▸), we assume pure *para*-hydrogen filling in our simulations, although the actual ratio under operational conditions is unknown.

### 3.2. Box pH_2_ moderator

#### 3.2.1. Implementation of pH_2_ box moderator at the BRR

**Figure 6:**
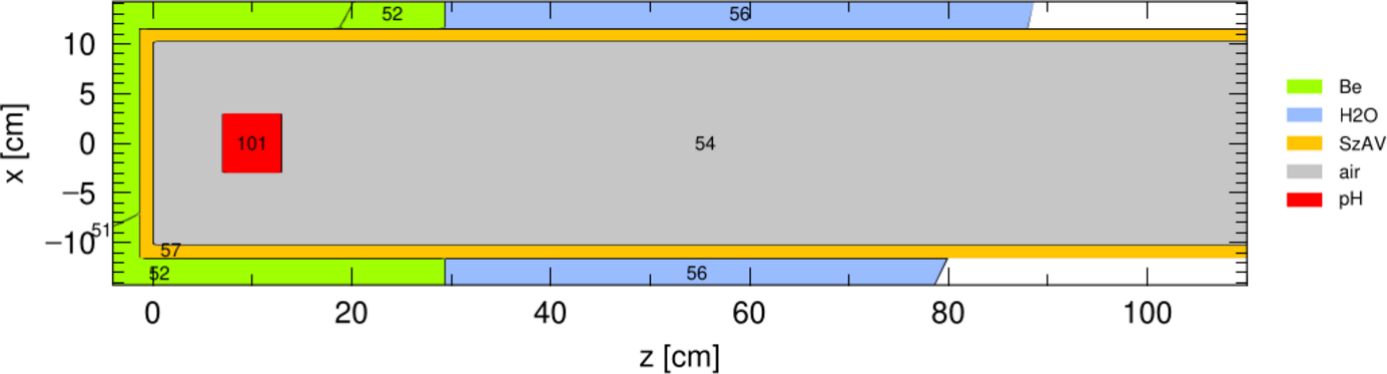
Top view of the moderator channel (cell 54) with a box pH_2_ moderator (cell 101).

The first scenario considered for improving the performance of the current disk moderator is a box pH_2_ moderator, depicted in Fig. 6 ▸. The box moderator is placed at the same distance of about 7 cm from the bottom of the channel as the existing disk moderator. In all subsequent simulations, the moderator is assumed to have 2 mm aluminium walls, which is considered technologically feasible.

The simulations are performed for two wavelengths: 2 Å (±10%) and 5 Å (±10%). These wavelengths are chosen for two reasons. First, while the majority of BRR instruments require 5 Å neutrons, some would still benefit from an increased flux of 2 Å neutrons. Second, neutrons at these wavelengths have different physical origins, as described in detail by Ioffe *et al.* (2025 ▸). In a large pH_2_ moderator (*e.g.* 10 × 10 × 10 cm), about half of the 5 Å neutrons are produced via a single-collision process, while the remainder originate from a multiple-collision process. For 2 Å neutrons, the single-collision process dominates.

This distinction is of significant practical importance. Neutrons produced through multiple collisions benefit from a larger moderator volume, as multiple scattering events require additional space. By contrast, the single-collision process occurs predominantly near the moderator walls and therefore benefits from compressing the moderator. Consequently, the resulting compression gain – the brightness increase achieved by reducing the moderator cross section – is expected to be greater for 2 Å neutrons, consistent with observations from ESS cold source simulations (Zanini *et al.*, 2019 ▸).

**Figure 7:**
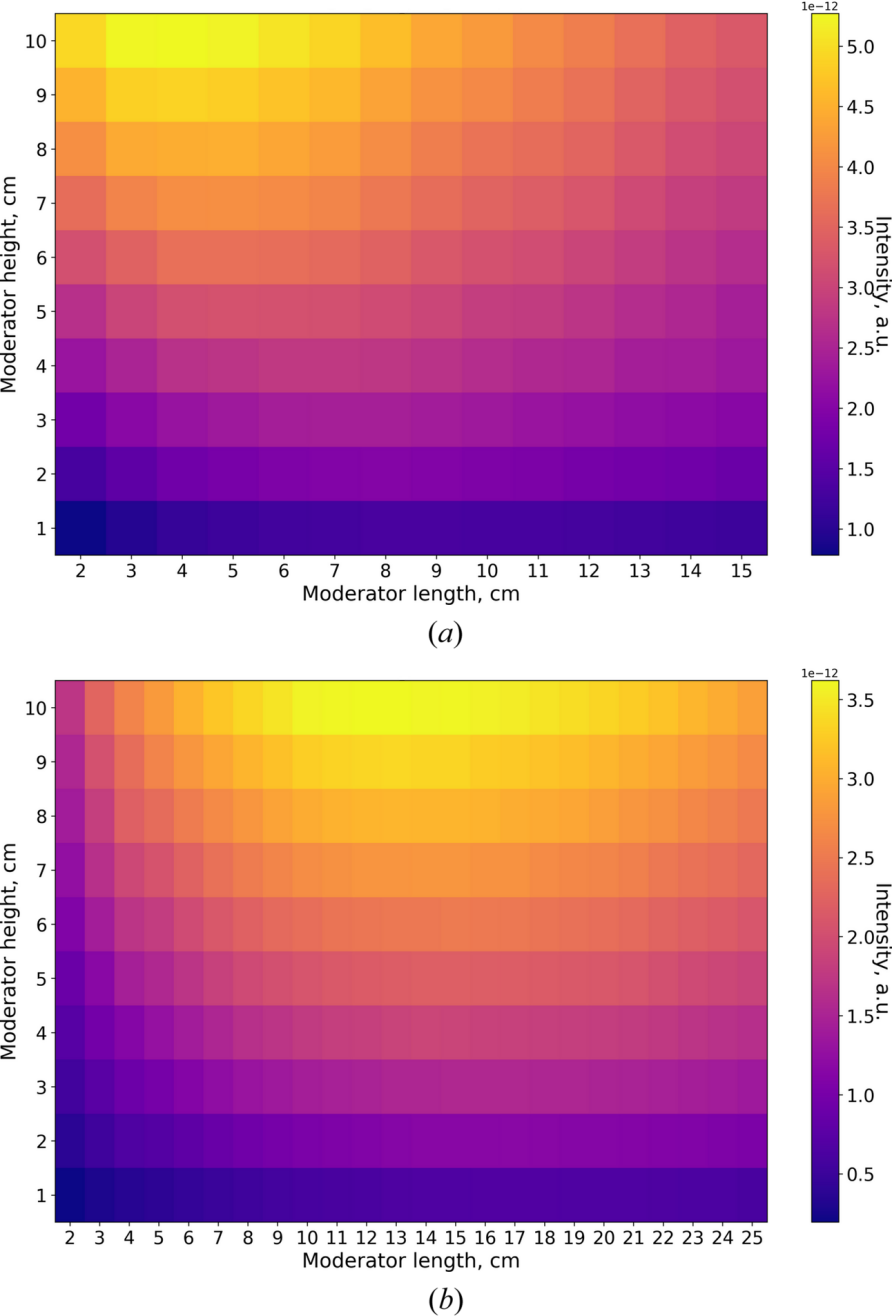
Box moderator intensity for two wavelengths, (*a*) 2 Å and (*b*) 5 Å.

The intensity calculations obtained for the box moderator are presented in Fig. 7 ▸. The moderator width was fixed at 10 cm, while the height and length were varied. In both wavelength bands, the maximum intensity occurs at the maximum height, although the optimal moderator length differs.

This difference arises from two competing effects. At small moderator lengths, the intensity increases with increasing length because the amount of *para*-hydrogen, where cold neutrons are produced, also increases. At the same time, the probability of cold neutron scattering or absorption along the path to the exit surface also grows. Under uniform thermal illumination, the brightness would eventually saturate: beyond a certain moderator length, only the region close to the exit surface contributes, while neutrons produced deeper in the moderator fail to reach the exit surface.

**Figure 8:**
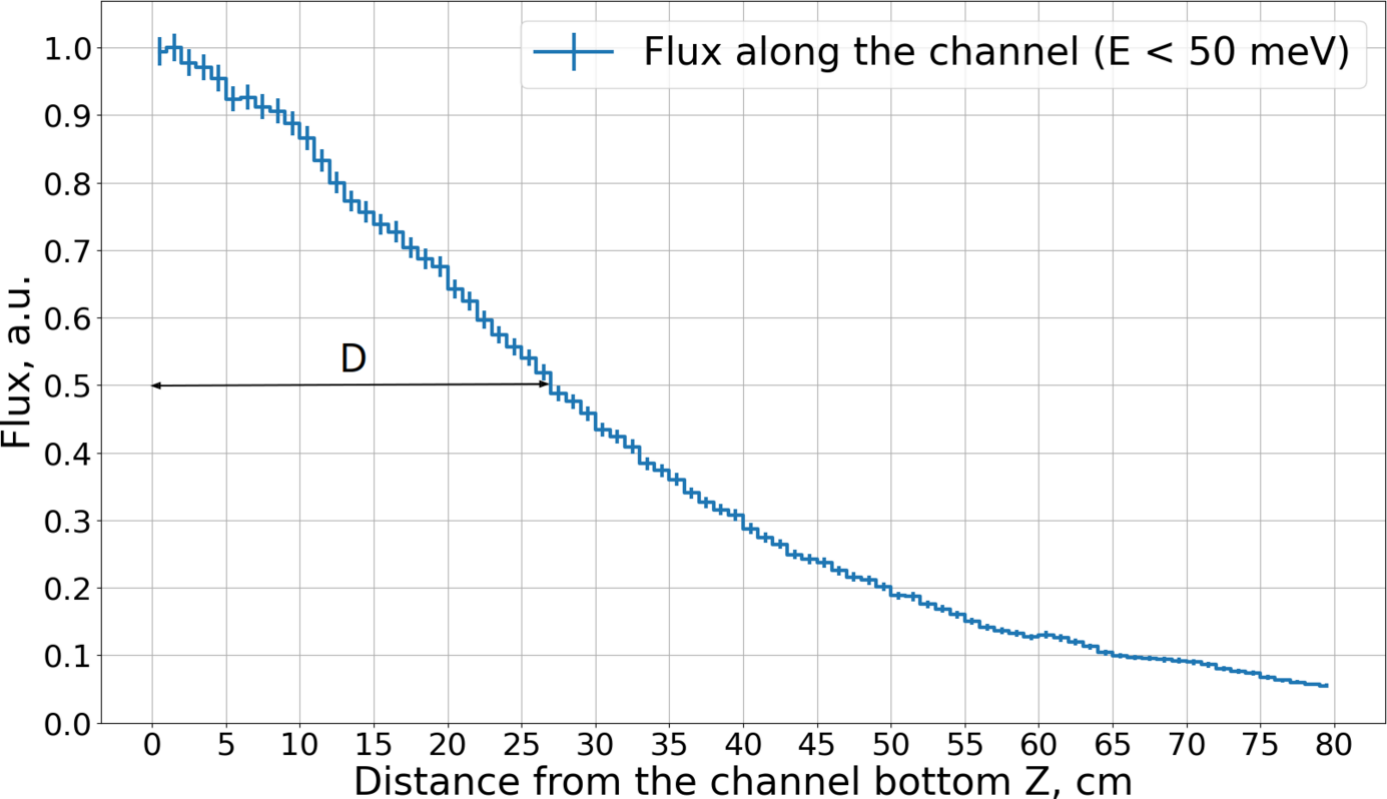
Neutron flux along the BRR moderator channel, integrated over the channel cross section and over energies up to 50 meV. *Z* is the axis along the channel (Fig. 6). *D* is the distance at which the flux decreases by half. Units are chosen in such a way that the maximum flux is unity.

In the case of the BRR geometry, however, the thermal illumination decreases along the moderator channel by half at about 27 cm from the bottom of the channel, as shown in Fig. 8 ▸. This further suppresses cold neutron production in longer moderators. Once scattering and absorption dominate over production, the brightness begins to decrease. The moderator length at which this transition occurs depends on the neutron cross section for the respective wavelength: for 2Å neutrons, the cross section is higher than for 5 Å neutrons (Fig. 1 ▸). As a result, the optimal moderator length is only 4 cm for 2 Å neutrons, in contrast to 13 cm for 5 Å neutrons.

**Figure 9:**
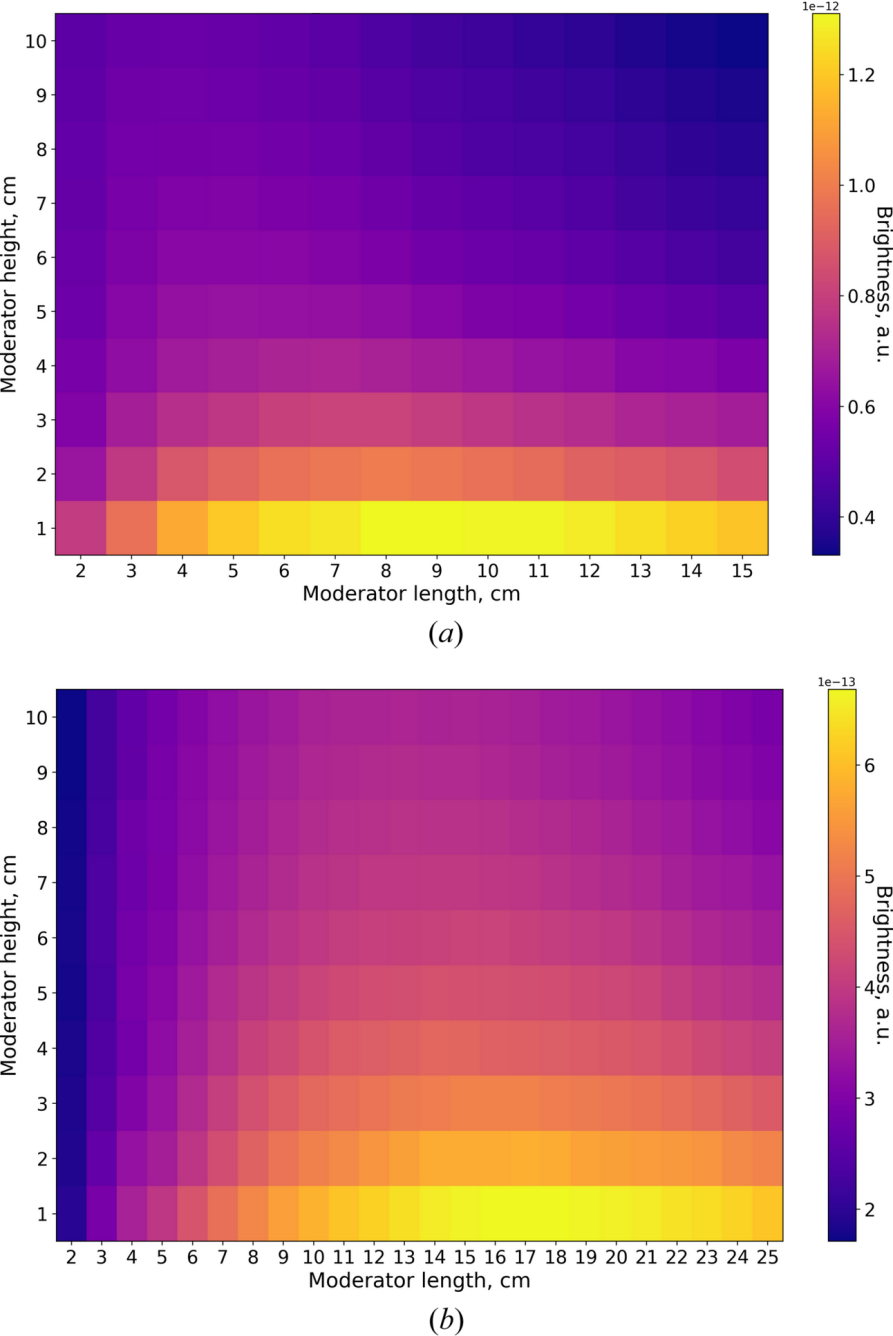
Box moderator brightness for two wavelengths, (*a*) 2 Å and (*b*) 5 Å.

Fig. 9 ▸ presents the same simulation results expressed as moderator brightness. The maximum brightness for both wavelengths is achieved at the minimum height of 1 cm. Again, the optimal lengths differ: 8–9 cm for 2 Å neutrons and 17 cm for 5 Å neutrons.

**Figure 10:**
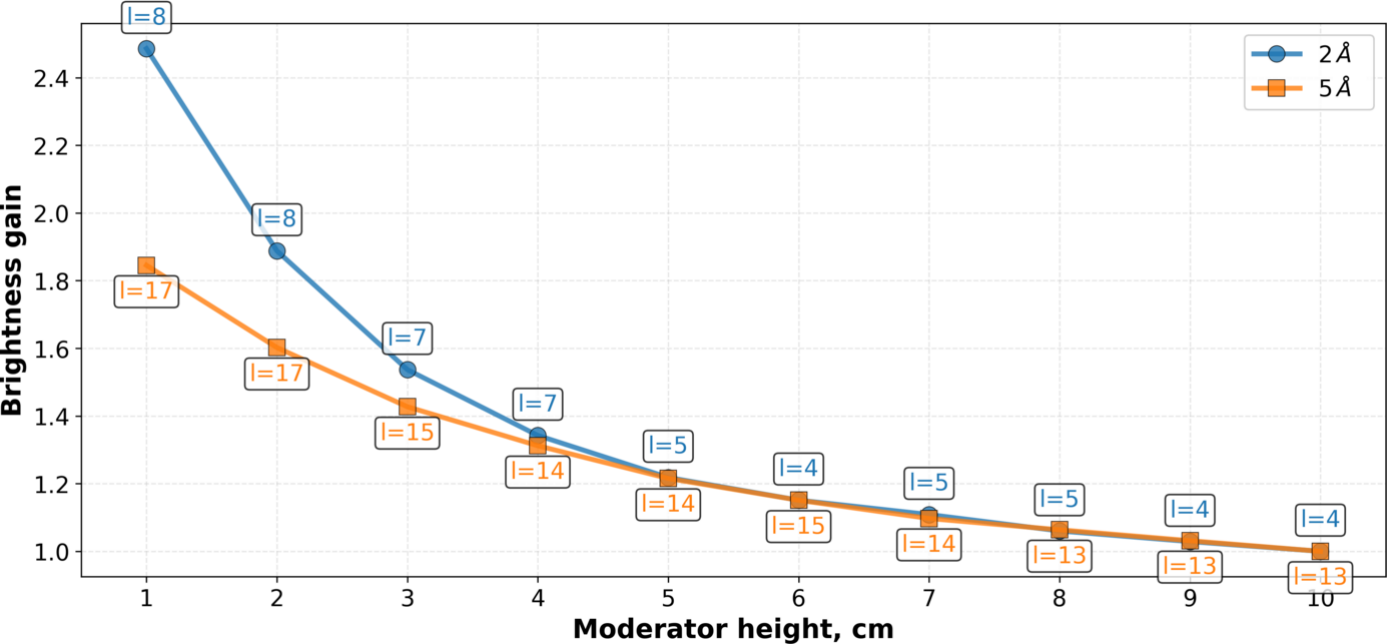
Box moderator brightness compression gain. For each height the optimal length is additionally shown. Units are chosen in such a way that the brightness at 10 cm is unity.

Moderator compression gain curves were subsequently constructed. For each moderator height in Fig. 9 ▸ the maximum brightness gain was selected. These curves are presented in Fig. 10 ▸, which also depicts the optimal moderator length for each height. As expected, the compression gains for 2 Å neutrons exceed those for 5 Å neutrons.

Both curves in Fig. 10 ▸ are monotonic, implying that the question of an optimal moderator size can only be answered by considering the specific requirements of the BRR instruments, as will be discussed in Section 3.2.2.

#### 3.2.2. Choice of box pH_2_ moderator width and height

The BRR hosts a diverse suite of cold neutron instruments, including two crystal monochromator reflectometers GINA (Bottyán *et al.*, 2013 ▸) and REF; the strain scanner ATHOS; small-angle scattering instruments Yellow Submarine (Almásy, 2021 ▸) and fSANS (Füzi & Rosta, 2010 ▸); and the multi-functional instrument PGAA, which combines various gamma activation and imaging techniques (Molnár *et al.*, 1997 ▸; Kis *et al.*, 2015*a* ▸). This suite will soon be complemented by the TOF spectrometer NEAT, relocated from the now-closed facility in Berlin (Russina *et al.*, 2018 ▸).

To define the required size of the cold moderator, the COFSI technique was employed, as described in detail by Konik & Ioffe (2023 ▸). This method allows one to translate any instrument’s phase space requirements (based on sample size and angular resolution) into the dependence of the optimal moderator size *D*_opt_ on the guide entrance size *w*_in_. Any moderator size *D*_m_ larger than *D*_opt_ for a particular *w*_in_ ensures full sample illumination given the appropriate neutron optics, whereas smaller moderators cannot provide full sample illumination.

In general, instruments demanding a larger phase space require a larger moderator. Among the BRR instruments, NEAT is the most demanding case, owing to its relatively large sample size (3–4 cm), the highest divergence (the focusing optics, a trumpet, is employed at the end of the guide) and its operation near 5 Å, which further increases the required moderator size for full guide entrance illumination. Therefore, the moderator width and height were selected exclusively on the basis of NEAT’s requirements; all other instruments are automatically accommodated.

**Figure 11:**
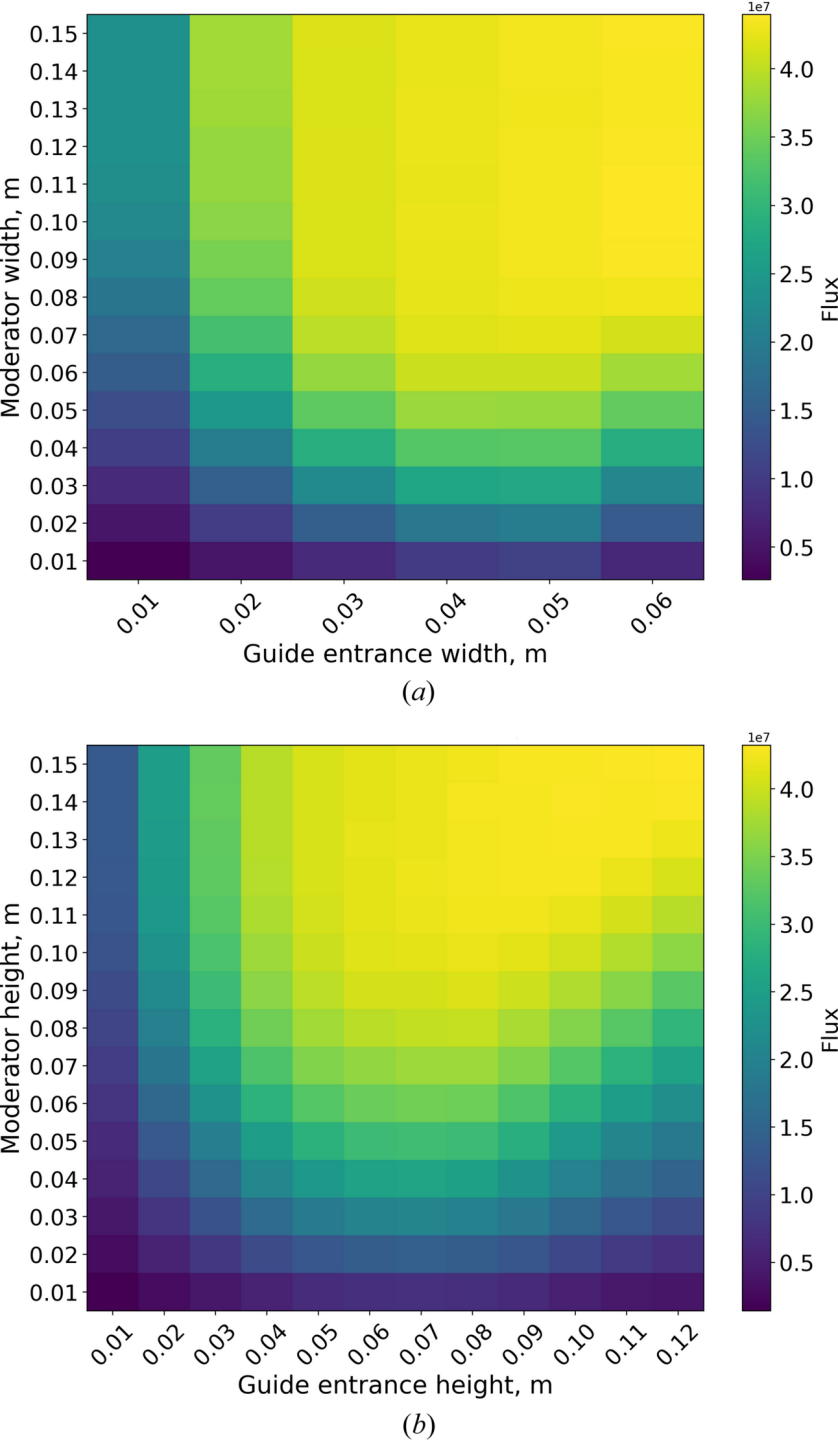
Sample flux maps calculated using *McStas* for the NEAT TOF spectrometer as a function of moderator size and guide entrance size, (*a*) width and (*b*) height.

Analytical COFSIs can be calculated, although they do not include assumptions about guide geometry or specific neutron optics. Because the upgrade strategy for NEAT involves reusing as much of the existing guide system as possible, a more accurate optics-aware determination was required. For this purpose, a series of Monte Carlo simulations were carried out using the *McStas* package (Willendrup & Lefmann, 2020 ▸; Willendrup & Lefmann, 2021 ▸). In these simulations, the existing guide described by Russina *et al.* (2018 ▸) is complemented by an adjustable anti-trumpet section starting 1.1 m away from the moderator. For each pair (*w*_in_; *D*_m_), the anti-trumpet length is optimized and the sample flux is determined. The resulting sample flux maps are presented in Fig. 11 ▸, with COFSIs corresponding to the envelopes of the yellow regions corresponding to maximum sample flux.

**Figure 12:**
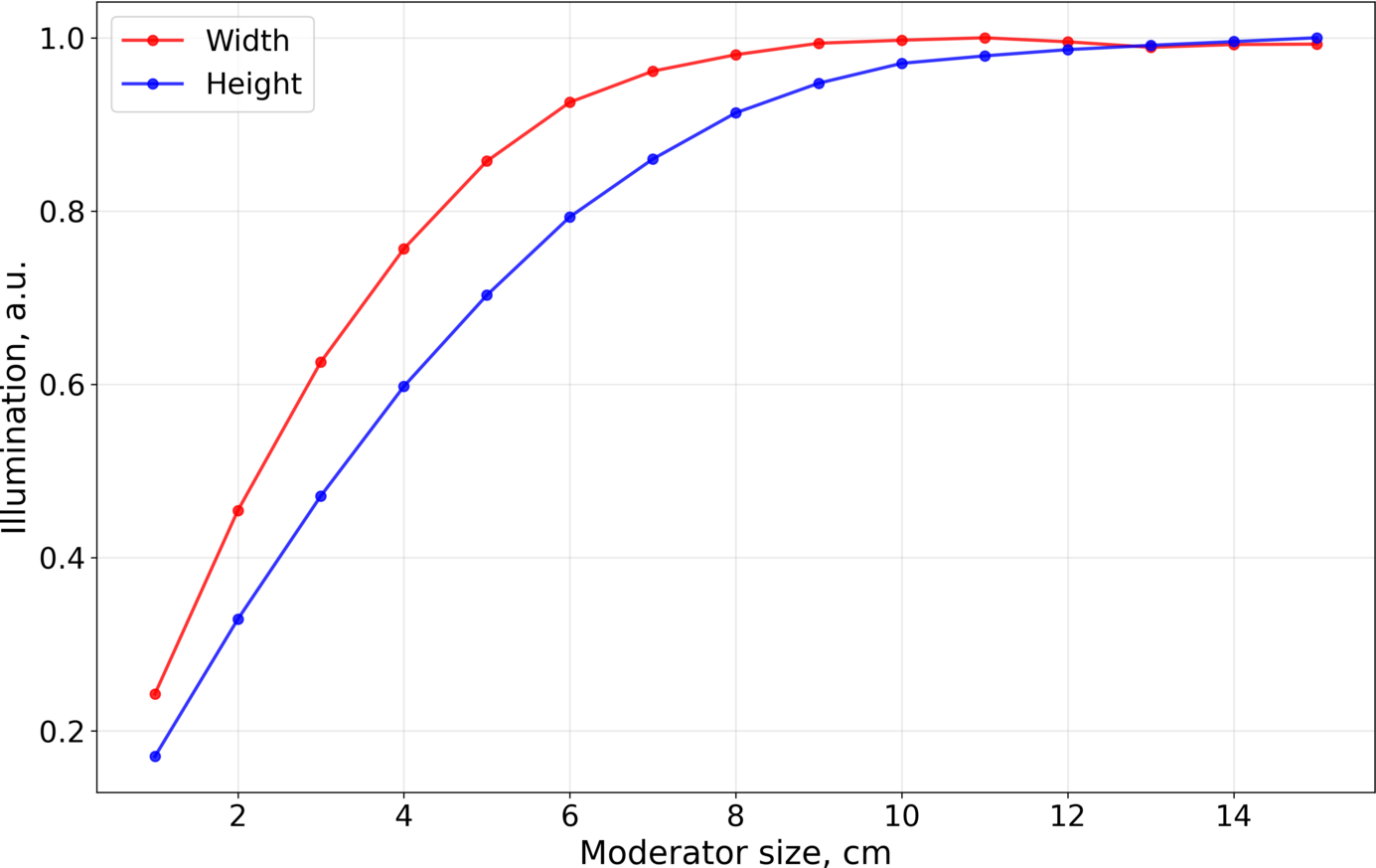
NEAT sample illumination curves as functions of moderator size. Units are chosen in such a way that the maximum illumination is unity.

These diagrams can also be interpreted as sample illumination maps if normalized to unity. In this representation (Fig. 12 ▸), the highest achievable illumination is identified for each moderator size, independent of guide entrance dimensions.

The curve corresponding to the horizontal dimension tends to lie above that for the vertical dimension. This difference arises from the NEAT phase space requirements being different in the horizontal and vertical directions: the sample size is 2 × 3 cm and the vertical divergence at the sample is larger than the horizontal divergence. In practical terms this means that the optimal moderator width can be smaller than the optimal height.

**Figure 13:**
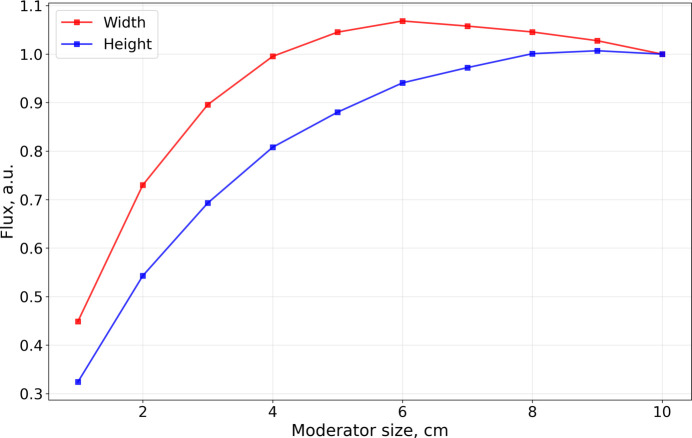
NEAT sample flux curves as functions of moderator size. Units are chosen in such a way that the flux at 10 cm is unity.

Note that the curves in Fig. 12 ▸ cannot be directly used to determine the optimal moderator dimensions, because they do not take into account the dependency of brightness on the moderator size, which is crucial for *para*-hydrogen (see Fig. 10 ▸). The sample flux as a function of moderator size can be obtained by simply multiplying the curves from Figs. 12 ▸ and 10 ▸, as illustrated in Fig. 13 ▸, from which the optimal moderator size can be identified.

The sample flux reaches a maximum at a moderator width of 6 cm and a height of 10 cm. However, a height of 6 cm is more favorable as it is consistent with the volume of *para*-hydrogen licensed for the BRR. Note that this height results in only 6% losses of the maximum flux (Fig. 13 ▸). The resulting 6 × 6 cm moderator face is used for all further optimization studies presented in this paper.

#### 3.2.3. Length optimization

**Figure 14:**
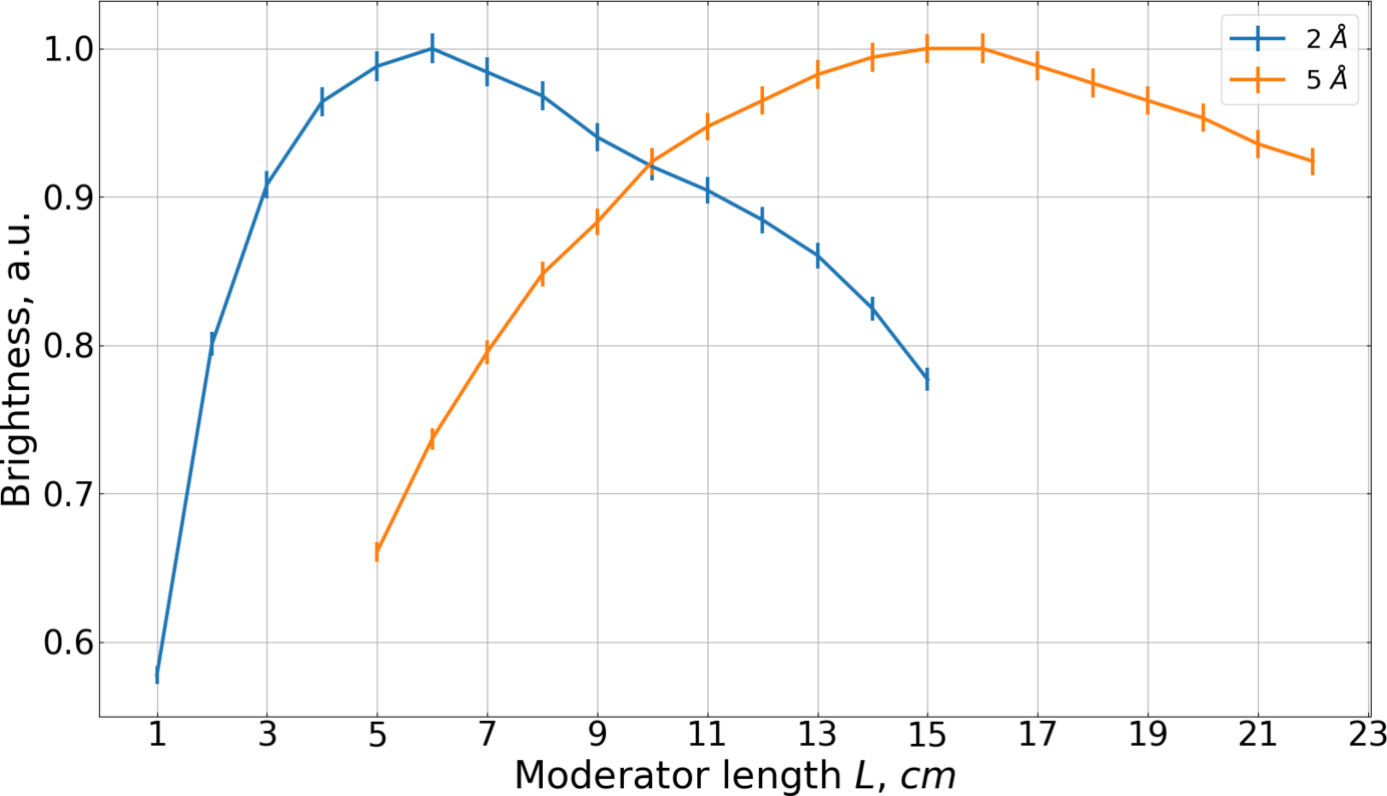
Dependence of the brightness of the box moderator on its length *L*. The blue curve corresponds to 2 Å and the orange curve to 5 Å. Units are chosen in such a way that the maximum brightness is unity.

With the moderator aperture fixed at 6 × 6 cm, the length *L* is varied to maximize its brightness. The results are shown in Fig. 14 ▸. The slight differences compared with the values obtained in Fig. 9 ▸ arise from the reduction in the moderator width from 10 to 6 cm.

### 3.3. Assemblies of flat *para*-hydrogen moderators

#### 3.3.1. Concept of assemblies

**Figure 15:**
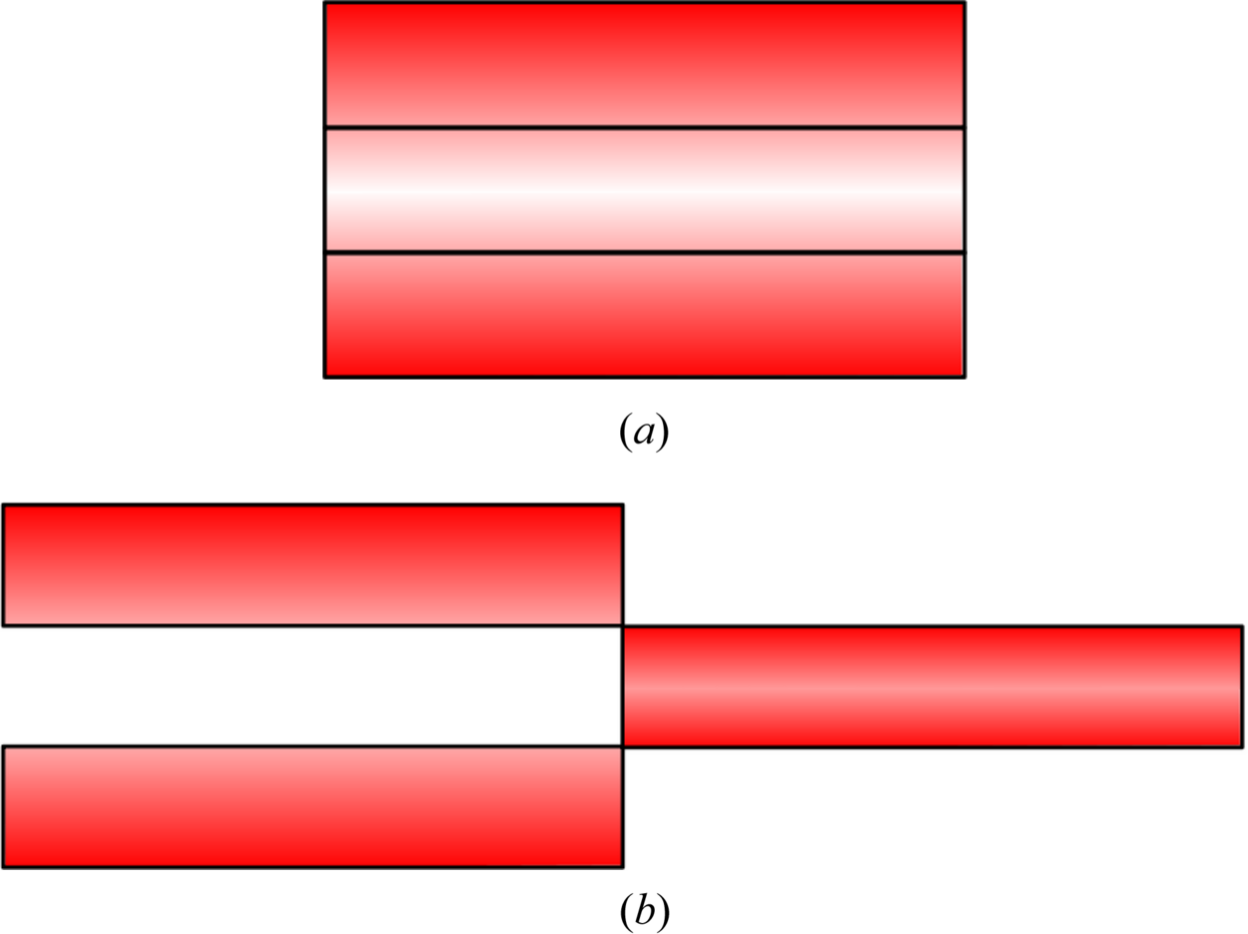
(*a*) Cold neutron production in a voluminous moderator. (*b*) Cold neutron production in an assembly. In a voluminous moderator the majority of cold neutron production (red gradient) occurs near the surface. To enhance the efficiency of the central region it is pulled outward.

The concept of assemblies of flat moderators has been proposed by Ioffe *et al.* (2025 ▸) and is illustrated in Fig. 15 ▸. The central part of the moderator, which contributes less to the overall brightness, is pulled outward to enhance performance. In this way, the high brightness of a flat moderator is reached, while the total moderator aperture is not changed. Since the distance to the detector is much larger than the size of this aperture, the gain in brightness directly translates into a gain in intensity.

**Figure 16:**
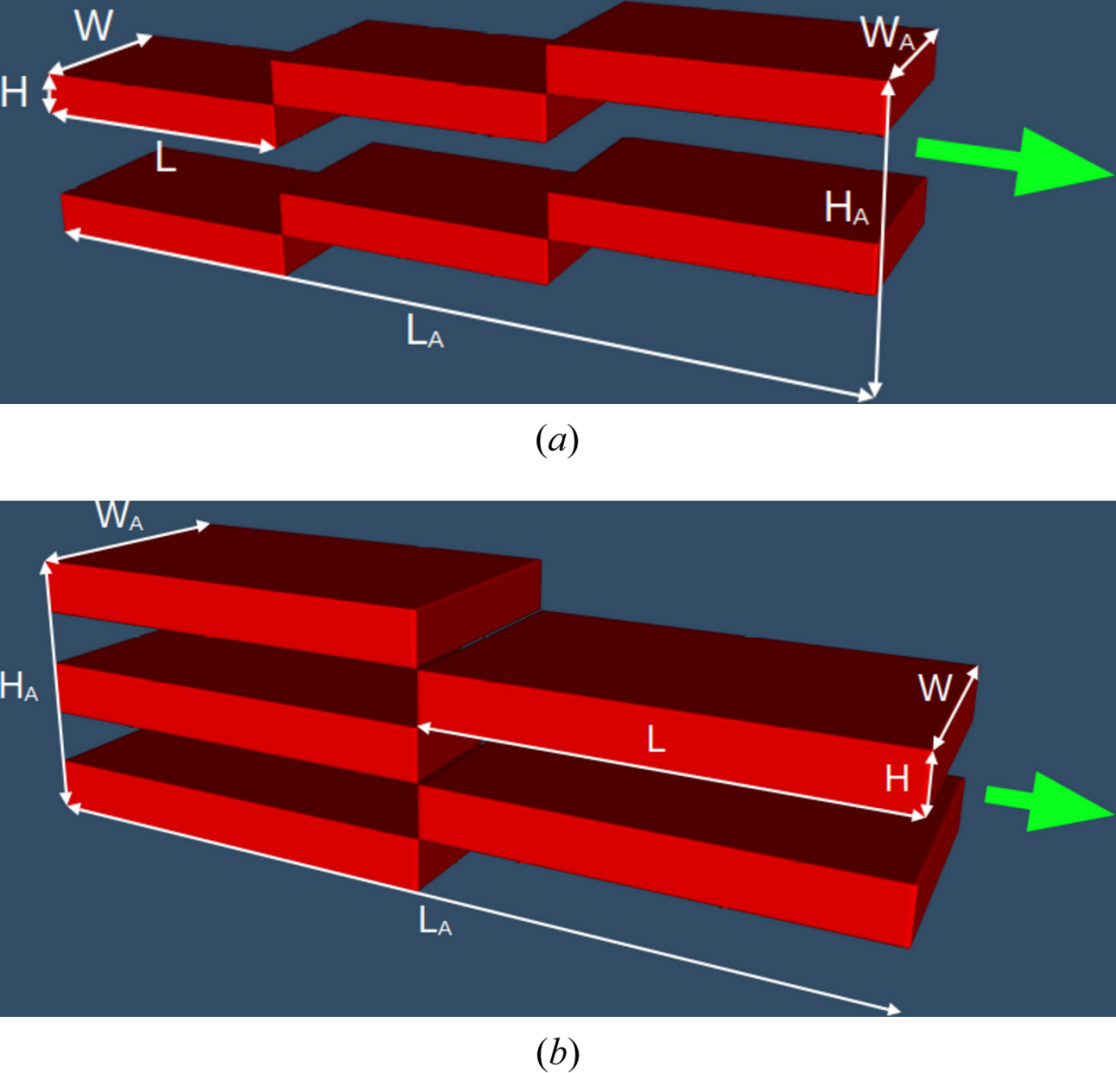
Examples of assemblies of flat *para*-hydrogen moderators. Green arrows indicate the direction of cold neutrons emitted from the moderator. (*a*) Two staircases with three steps each. In this example *H*_A_ = 6*H*, *L*_A_ = 3*L* and *W*_A_ = *W*. (*b*) Five-step chessboard. In this example *H*_A_ = 5*H*, *L*_A_ = 2*L* and *W*_A_ = *W*.

We distinguish two types of assemblies, ‘staircase’ and ‘chessboard’ configurations. Each assembly consists of several flat moderator elements or ‘steps’. The dimensions are defined as follows: width *W*, height *H* and length *L* for the step, and corresponding values *W*_A_, *H*_A_ and *L*_A_ for the whole assembly (Fig. 16 ▸).

In the staircase configuration, steps are sequentially attached to each other at their corners, forming an extended structure. Multiple staircase units may be stacked vertically. A staircase consisting of only one step corresponds to the box moderator considered above. An example of an assembly composed of two staircases, each containing three steps, is shown in Fig. 16 ▸(*a*).

In a chessboard configuration, a series of steps are arranged in a chessboard pattern, so that the total length of the assembly *L*_A_ corresponds to twice the length of a single step *L*. A chessboard with an even number of steps is equivalent to an assembly of staircases with two steps. Therefore, we consider only chessboards with an odd number of steps. An example of a five-step chessboard is shown in Fig. 16 ▸(*b*).

**Figure 17:**
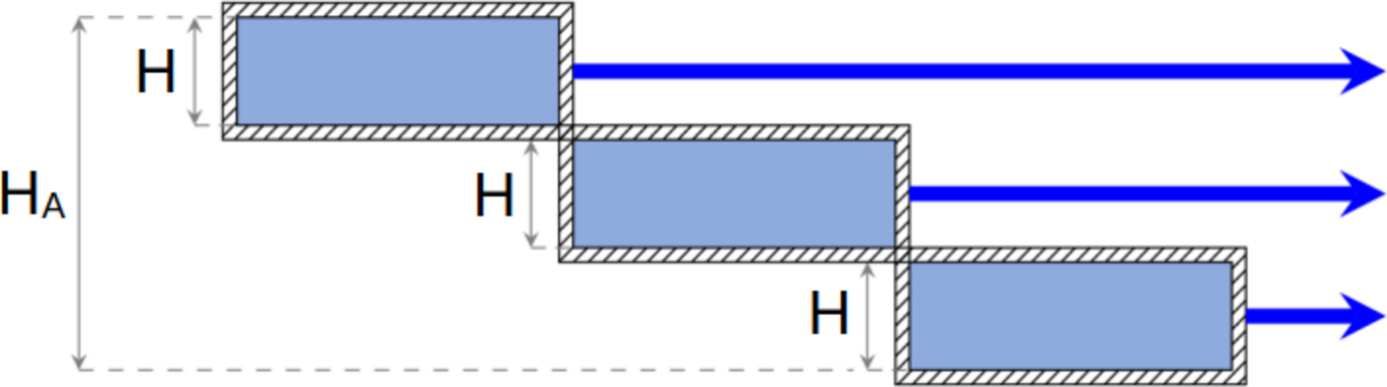
Scheme of the assembly with walls. Blue arrows indicate the direction of cold neutrons emitted from the moderator. Adapted from Ioffe *et al.* (2025 ▸).

**Figure 18:**
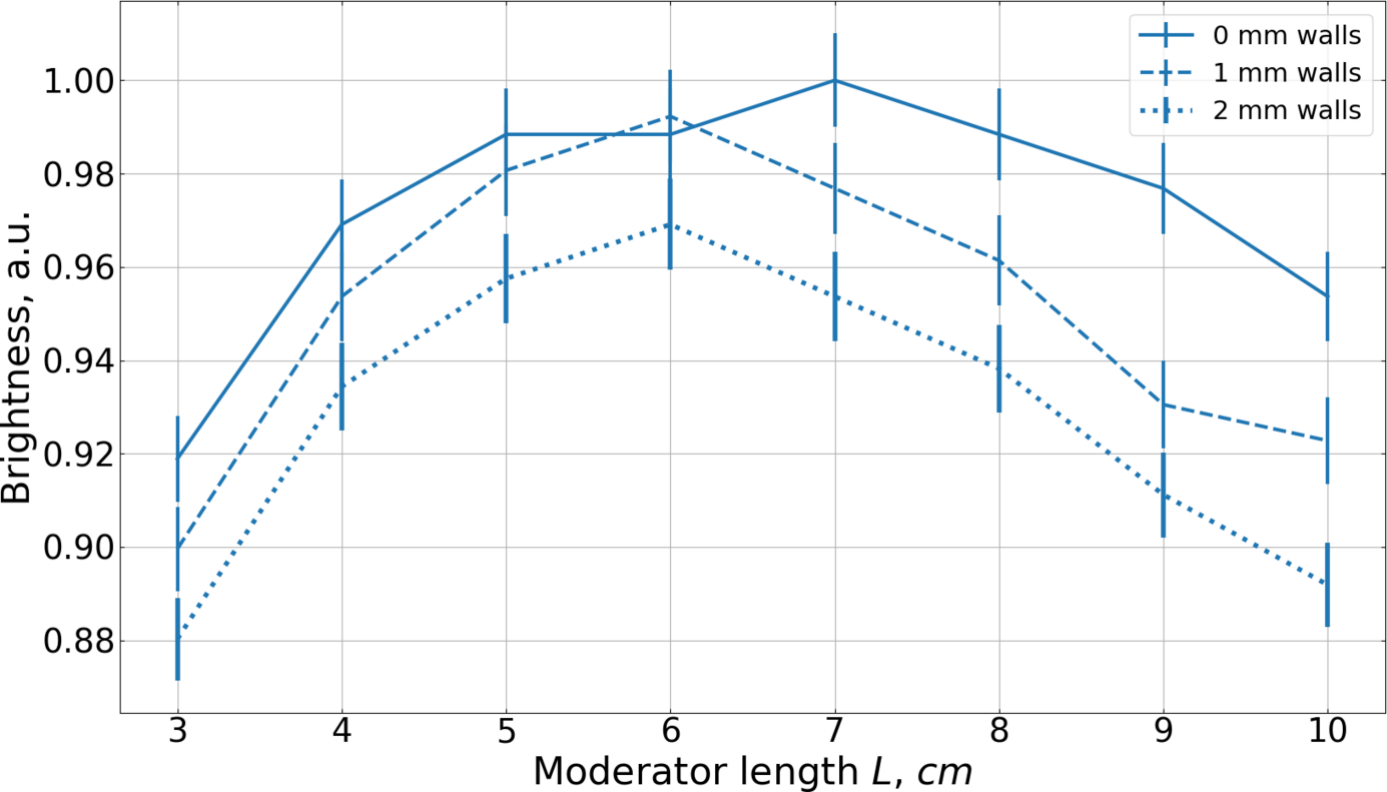
Dependence of the brightness of the box moderator on its length *L* for 2 Å neutrons with wall thicknesses of 0 mm (solid line), 1 mm (dashed line) and 2 mm (dotted line). Units are chosen in such a way that the maximum brightness is unity.

**Figure 19:**
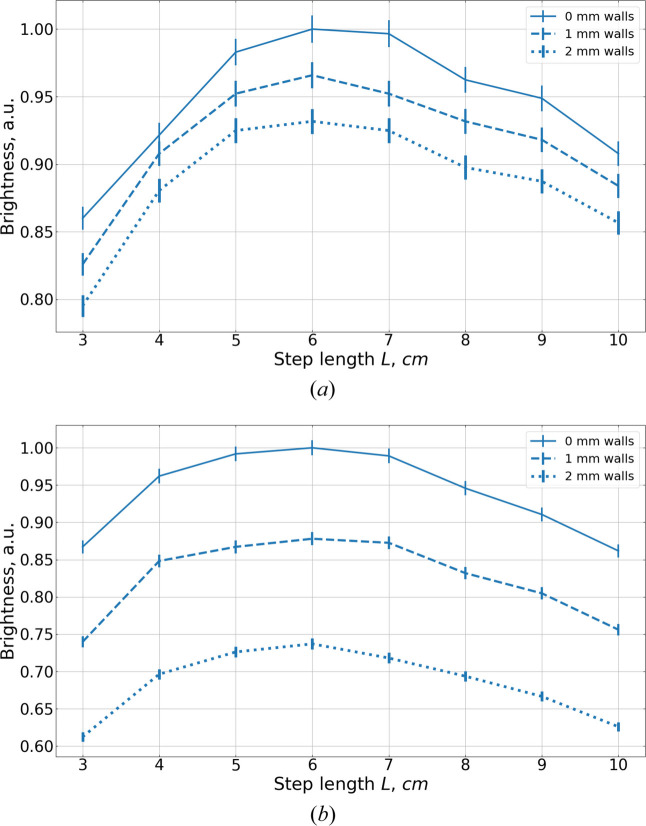
Dependence of the brightness of the moderator assembly on its step length *L* for 2 Å neutrons with wall thicknesses of 0 mm (solid line), 1 mm (dashed line) and 2 mm (dotted line). Units are chosen in such a way that the maximum brightness is unity. (*a*) One staircase made of two steps and (*b*) four staircases made of three steps.

The walls of the assemblies are made of 2 mm thick aluminium, arranged as shown in Fig. 17 ▸. They reduce the moderator performance in two ways. First, thermal neutrons arriving at shallow angles traverse relatively long paths through the aluminium, leading to significant absorption and, consequently, a reduction in cold neutron production. This behavior is demonstrated in Fig. 18 ▸, where the brightness of a box moderator is plotted for different wall thicknesses. Second, because the overall moderator height *H*_A_ is fixed, the introduction of walls comes at the cost of the *para*-hydrogen height *H*. The more steps or staircases there are in the assembly, the more aluminium layers there are between *para*-hydrogen cells. For example, a single staircase, composed of two steps, contains only a single aluminium layer, and the impact of a finite wall thickness is minor [Fig. 19 ▸(*a*)]. However, the assembly of four staircases with three steps each contains eleven aluminium layers, leading to a much more pronounced reduction in brightness gain [Fig. 19 ▸(*b*)].

Another feature of the assembly is that each step in it performs worse than an identical step placed alone. This reduction arises from mutual screening of the steps from thermal neutrons, an effect we refer to as ‘shadowing’. If neighboring steps are absolutely transparent, the shadowing is 0, while complete blockage of thermal illumination corresponds to 100% shadowing. The presence of shadowing implies that, in principle, the number of steps in an assembly should be minimized.

Both the effect of finite wall thickness and the shadowing effect are naturally taken into account in our Monte Carlo simulations.

#### 3.3.2. Moderator assemblies at BRR

The performance of the assemblies of flat *para*-hydrogen moderators at the BRR is evaluated using our *PHITS* model. The total aperture of 6 × 6 cm and 7 cm distance to the bottom of the moderator channel are kept consistent with the box moderator configuration. The same neutron wavelengths of 2 Å (±10%) and 5 Å (±10%) are examined.

The moderator brightness is maximized by varying the step length *L*. The results are normalized to the maximum brightness of a box moderator (one staircase with one step), yielding brightness gains *g* = *B*_1_/*B*_2_, where *B*_1_ is the brightness of the assembly and *B*_2_ is the maximum brightness of the box moderator. Note that, in this configuration, the brightness gain is equivalent to the flux gain.

**Figure 20:**
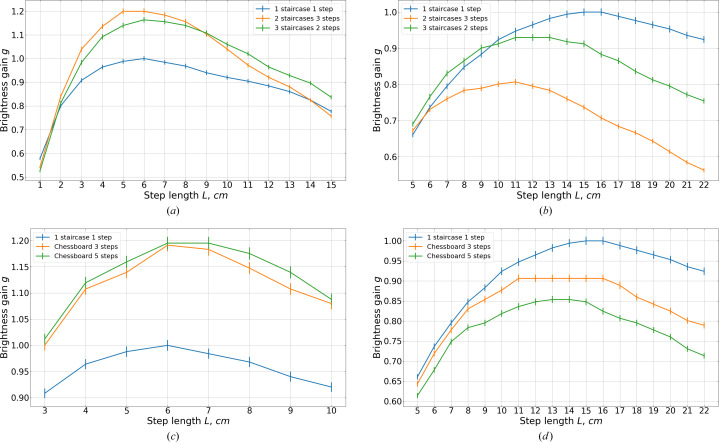
Brightness gains of the assemblies with the best observed performance, (*a*) 2 Å staircases, (*b*) 5 Å staircases, (*c*) 2 Å chessboards and (*d*) 5 Å chessboards. In each subfigure the gains are calculated relative to the corresponding optimal box moderator (one staircase with one step).

We investigated assemblies composed of one, two, three and four staircases, with two and three steps each, as well as chessboard assemblies made of three, five, seven and nine steps. No further performance improvement was observed with additional staircases or steps. Fig. 20 ▸ presents the results of the geometries that yield the highest brightness gains *g* for each wavelength.

For 2 Å neutrons the maximum gain of approximately 1.2 is reached with a step length *L* = 6 cm for both the assembly composed of two staircases with three steps [orange line in Fig. 20 ▸(*a*)] and the chessboard with three or five steps [orange and green lines in Fig. 20 ▸(*c*)]. However, such a configuration, optimized for 2 Å neutrons, results in a 20−25% reduction in gain for 5 Å neutrons compared with a geometry optimized for 5 Å neutrons [orange line in Fig. 20 ▸(*b*), and orange and green lines in Fig. 20 ▸(*d*) at *L* = 6 cm].

For 5 Å neutrons all assemblies perform worse than the box moderator, achieving at best a gain of ∼0.9 (*i.e.* a 10% loss), obtained with three staircases made of two steps [green line in Fig. 20 ▸(*b*)] and with the chessboard made of three steps [orange line in Fig. 20 ▸(*d*)].

### 3.4. Discussion of the results

#### 3.4.1. Box moderator

Two box moderators are identified as optimal: a shorter design optimized for the production of 2 Å neutrons, and a longer design optimized for 5 Å neutrons.

**Figure 21:**
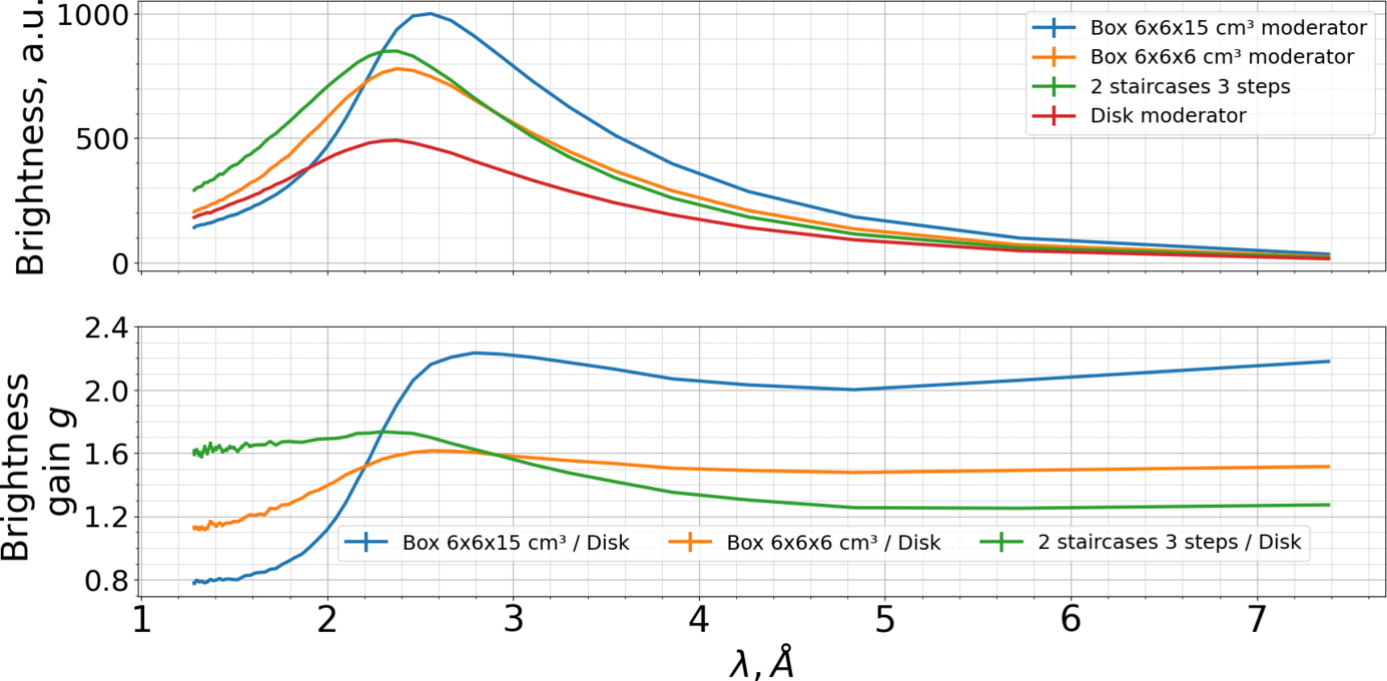
(Top) Spectra of 5 Å-optimized 15 cm long box moderator (blue line), 2 Å-optimized 6 cm long box moderator (orange line), 2 Å-optimized assembly made of two staircases with three steps each (green line) and the current disk moderator (red line). Units are chosen in such a way that the minimum brightness of the disk moderator is unity. (Bottom) Brightness gains *g* of the new box moderators (15 cm long box — blue line, 6 cm long box — orange line) and of the assembly relative to the current disk moderator

Their spectra, along with that of the current disk moderator, are shown in Fig. 21 ▸. The shorter box moderator demonstrates better performance than the current disk moderator across the entire wavelength range, with brightness gains between 1.2 and 1.6.

In contrast, the longer box moderator outperforms the current moderator only for λ > 2 Å. However, for cold neutrons with λ > 2.5 Å its gains are much higher, ranging from 2 to 2.2. The longer moderator thus surpasses the shorter one for all wavelengths longer than 2.3 Å.

#### 3.4.2. Assembly

Previous studies (Ioffe *et al.*, 2025 ▸) suggested that assemblies of flat moderators may provide additional gains over box moderators. For the BRR geometry, however, the results shown in Fig. 20 ▸ indicate that assemblies do not perform well for 5 Å neutrons, although they still provide noticeable brightness gains for 2 Å neutrons.

This difference originates from two independent factors governing the performance of an assembly:

(i) Compression gains: More compressed steps generally produce higher brightness. As demonstrated in Fig. 10 ▸, compression gains are significantly larger for 2 Å neutrons than for 5 Å neutrons.

(ii) Thermal neutron distribution along the channel: Efficient operation requires that several steps are placed within regions of sufficiently high thermal flux. Since the optimal steps for 2 Å neutron production are considerably shorter (see *e.g.* Fig. 14 ▸), multiple steps can be positioned within the high-illumination zone. For 5 Å neutrons the optimal steps are much longer, making this condition harder to satisfy. For more detailed discussion of the influence of thermal flux distribution on the assembly performance, see Appendix *B*[App appb].

In the BRR configuration, both factors favor the performance of 2 Å-optimized assemblies and simultaneously suppress the performance of those optimized for 5 Å neutrons.

As shown in Fig. 21 ▸, the 2 Å-optimized staircase assembly indeed yields higher brightness than the corresponding box moderator optimized for the same wavelength in the range λ < 3 Å (green curve). For λ > 3 Å the staircase moderator is less efficient than the box one, although it still achieves modest gains relative to the current disk moderator. Note that for the 6 cm high two-staircase three-step assembly with 2 mm walls the total height of *para*-hydrogen becomes 5 cm.

## 4. Conclusion

The forthcoming modernization of the Budapest Research Reactor provides an opportunity to replace the existing cold neutron source with a more efficient one. In recent years, cold moderators based on liquid *para*-hydrogen have been shown to offer the highest performance. In particular, low-dimensional (flat) *para*-hydrogen moderators have attracted considerable interest due to their potential for significantly increased brightness. In this work, the performance of such moderators has been analyzed under the specific conditions of the BRR reactor.

It was found that replacing the existing disk *para*-hydrogen moderator (diameter 12 cm, thickness 4 cm) with a box *para*-hydrogen moderator of size 6 × 6 × 15 cm leads to a 2–2.2 times gain in the brightness of the outgoing cold neutron beam with wavelengths in the range 3–7 Å. The cross section of the moderator is determined according to the requirements of the BRR instrumentation suite.

The achievable gain is constrained by the neutron characteristics of the reactor channel. Specifically, a relatively short flux-dense region (about 20 cm along the channel axis) and the strong non-uniformity of the thermal neutron flux distribution prevent full utilization of the main advantage of *para*-hydrogen — the large mean free path of cold neutrons, which enables intensity accumulation along a long moderator.

Given the potential of staircase moderators to enhance brightness and intensity further (Ioffe *et al.*, 2025 ▸), their performance was also analyzed as a modification of the box *para*-hydrogen moderator installed within the same reactor channel. While an additional gain of about 20% was obtained for 2 Å neutrons, no noticeable improvement was observed for wavelengths of 5 Å and above. This behavior can be attributed to two factors: First, the compression gains for 2 Å neutrons are larger than those for 5 Å neutrons (see Fig. 10 ▸). Second, it reflects the thermal neutron illumination pattern of the cold moderator; the efficiency of *para*-hydrogen moderators depends on neutron wavelength, with the optimal moderator length increasing for longer wavelengths.

Overall, the presented work demonstrates how instrument requirements and the choice of desirable neutron wavelength influence the design of cold neutron moderators. The obtained results provide a solid foundation for the final optimization of the future BRR cold neutron source.

## Figures and Tables

**Figure 22:**
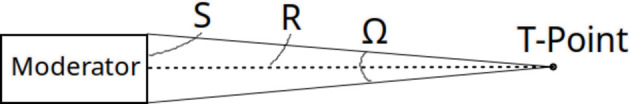
Measurement scheme in *PHITS*.

**Figure 23:**
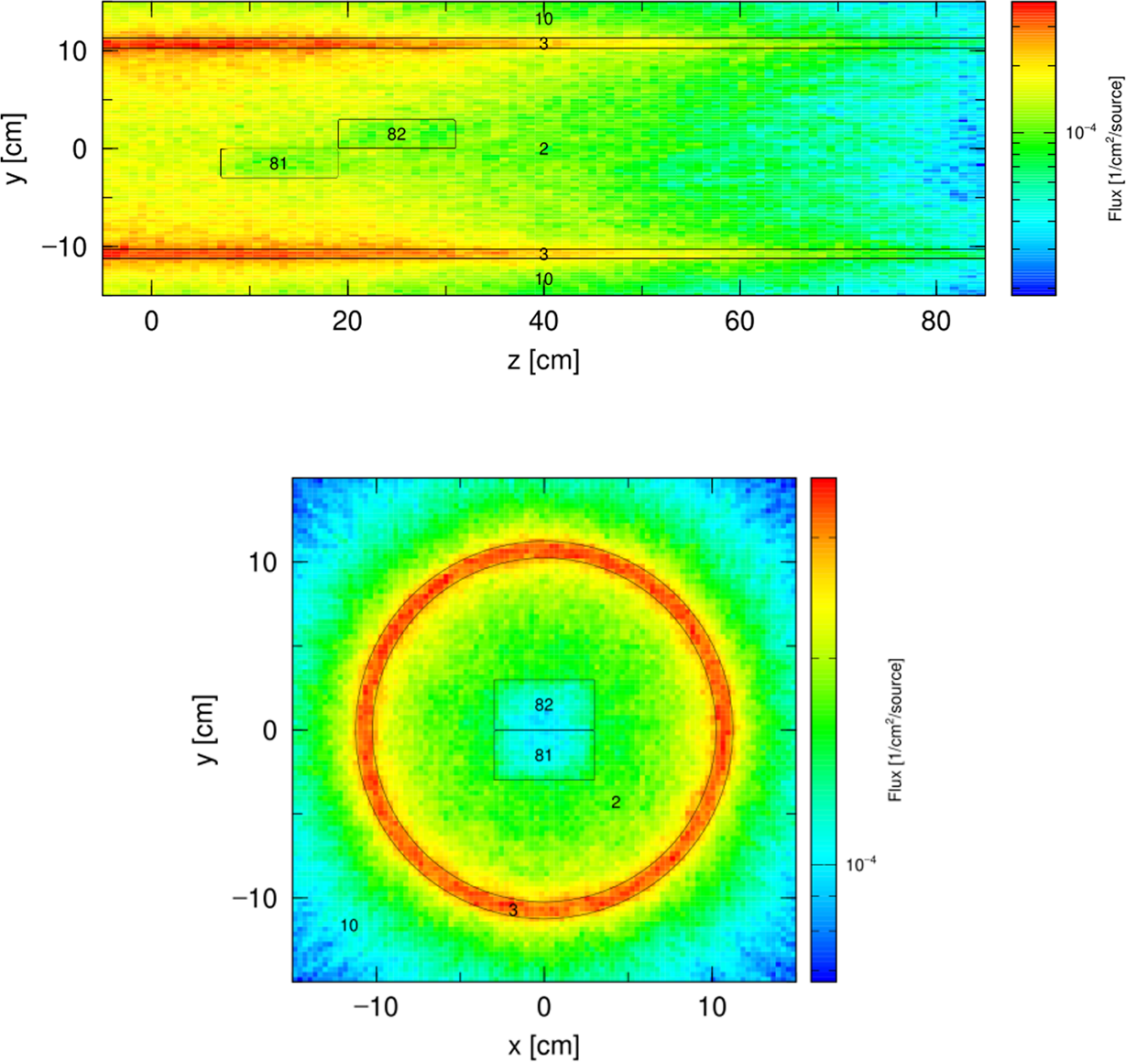
Cross cuts of the model channel, (top) along and (bottom) perpendicular to the channel axis. The neutron source, defined by a Gaussian distribution along the channel axis, is placed at the surface of the channel.

**Figure 24:**
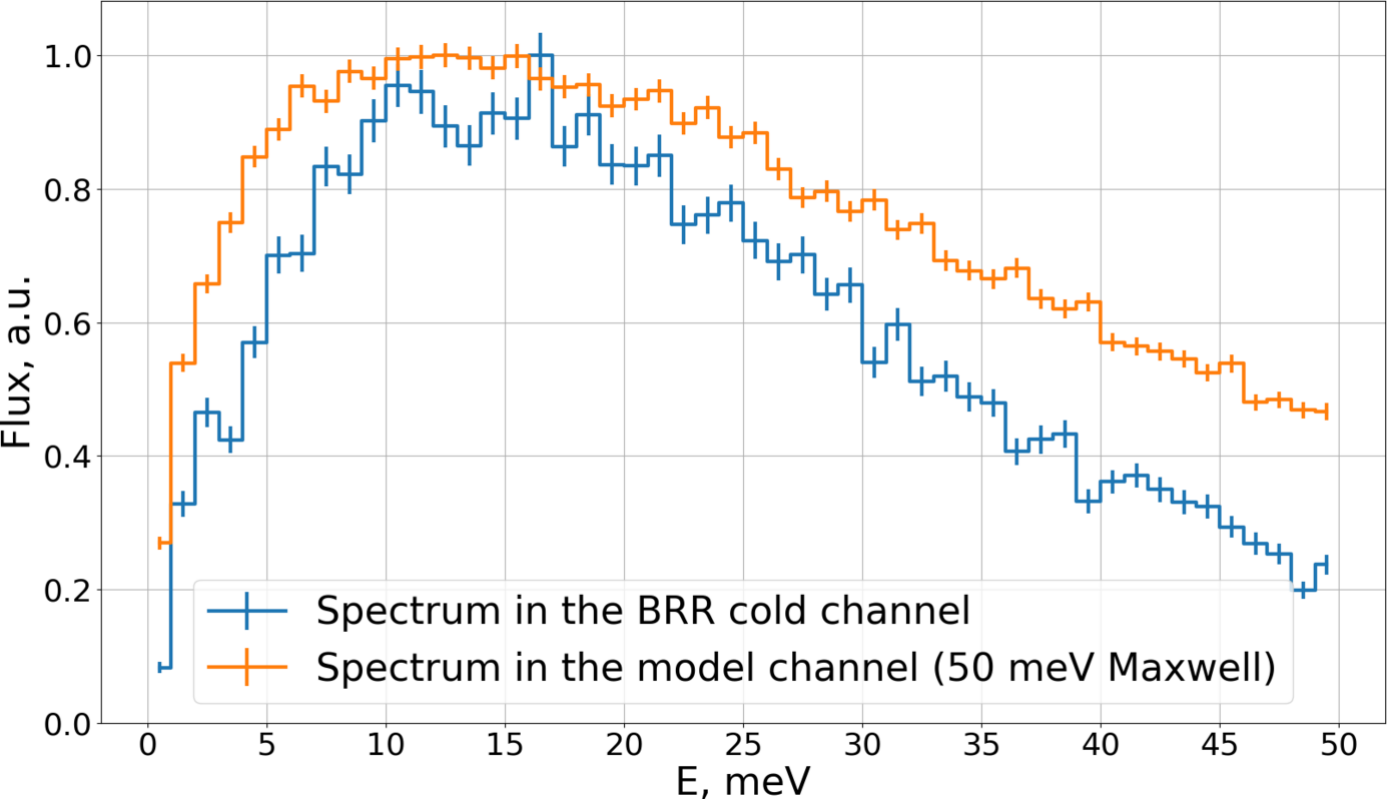
Neutron energy spectrum inside the BRR moderator channel [cell 54 in Fig. 3(*a*)] (blue line) and inside the model channel (orange line). Flux is integrated over the whole channel. Units are chosen in such a way that the maximum flux is unity.

**Figure 25:**
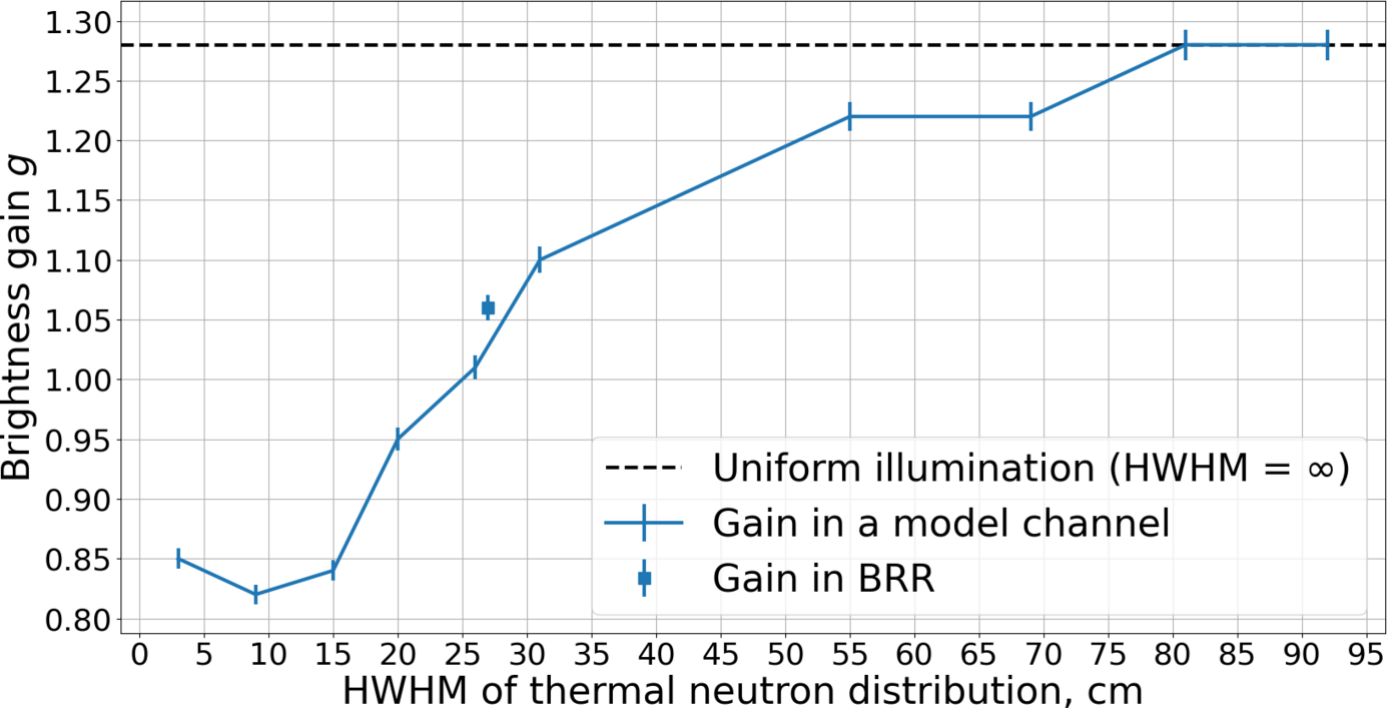
Brightness gain against the illumination width.

## Data Availability

The data will be available upon request.

## Appendix A Figure of merit: brightness

A typical measurement scheme in *PHITS* is shown in Fig. 22 ▸. A point detector T-Point is placed at a distance *R*, which is much greater than the moderator size. The exit surface area of the moderator is *S*, so the solid angle Ω under which the detector sees the moderator can be approximated as

The quantity measured by a detector T-Point is the neutron flux Φ,

where the energy and time integration are taken over the ranges specified for the detector, and the solid angle integration is over the full 4π. Here *v* is the neutron speed and ρ is the neutron density, defined as

*i.e.* the number of neutrons *N* per unit volume d*V*, per solid angle dΩ, per energy d*E*.

Since neutrons reach the detector only within a solid angle Ω, we define the brightness *B* as

Strictly speaking, *B* is the energy- and time-integrated brightness, while the ‘true’ brightness *B*_0_ is usually defined as (Zanini *et al.*, 2019 ▸)

so that the flux Φ is the brightness *B*_0_ integrated over energy, time and solid angle, as follows from equation (5).

In this work we are often interested in the relative brightness of one moderator over another, which we define as the brightness gain *g*:

Here, *B*_1_ and *B*_2_ are the brightnesses of the two moderators. Since we calculate *B*_1_ and *B*_2_ with the same detector under identical conditions, it makes no difference whether we use *B* or *B*_0_ from the point of view of equation (9).

Note that if the moderators have the same aperture *S*, the brightness gain *g* is also the flux gain.

Throughout this paper, when moderator geometries with different aperture sizes are compared, it is implicitly assumed that the instrument views the entire moderator exit surface in all cases. Consequently, the phase space region sampled by the instrument changes when the moderator aperture changes. This comparison therefore assumes that the neutron brightness is uniform across the moderator exit surface, such that variations in the sampled phase space volume associated with different moderator apertures do not affect the reported brightness values.

For moderators with non-uniform brightness distributions (*e.g.**para*-hydrogen moderators), a more rigorous comparison can be achieved by evaluating the brightness over a fixed phase space volume corresponding to the instrument acceptance, as implemented in recently developed tools such as *Brightify* (Akhyani *et al.*, 2026 ▸; https://github.com/BrightnessTools/Brightify).

## Appendix B Influence of thermal neutron flux distribution

The space for building an effective assembly of moderators is severely limited in a rapidly decreasing thermal neutron distribution along the channel axis.

To demonstrate this effect we have created a model channel in *PHITS* with a varying width of neutron distribution along the channel. The channel is a cylinder of the same radius as the BRR moderator channel. The source is simulated by a Gaussian distribution placed at the surface of the channel (Fig. 23 ▸) with varying half-width at half-maximum (HWHM) along the channel axis. Its center is located at the bottom of the BRR moderator channel, so HWHM is the distance to the bottom at which the distribution drops down by half (it corresponds to *D* in Fig. 8 ▸). The energy spectrum of the source is defined by a Maxwellian distribution, which simulates the spectrum inside the BRR moderator channel (Fig. 24 ▸).

The cold source is one staircase made of two steps filled with *para*-hydrogen (Fig. 23 ▸). It is placed at 7 cm from the center of a Gaussian distribution, which represents the distance to the bottom of the BRR moderator channel. The moderator’s aperture of 6 × 6  cm is the same as that used in the BRR model and the step length *L* is 12 cm.

We calculate the brightness gain *g* of the staircase relative to a box moderator of the same volume for a neutron wavelength of 2 Å (±10%) depending on the HWHM of thermal illumination along the channel. The results are presented in Fig. 25 ▸. The brightness gain is less than 1 (which means the absence of gain) up to a distribution width of about 25 cm. If the distribution is wide enough to be considered uniform (HWHM > 80 cm), the brightness gain saturates and reaches a value of about 1.28. If the distribution width in the model channel corresponds to that in the BRR moderator channel, we observe a good agreement between the respective values of *g*.
